# A Descriptive Study on Causes of Death in Hospitalized Patients in an Acute General Hospital of Southern Italy during the Lockdown due to Covid-19 Outbreak

**DOI:** 10.3390/healthcare9020119

**Published:** 2021-01-25

**Authors:** Pasquale Mascolo, Alessandro Feola, Carmen Sementa, Sebastiano Leone, Pierluca Zangani, Bruno Della Pietra, Carlo Pietro Campobasso

**Affiliations:** 1 Department of Experimental Medicine, University of Campania “Luigi Vanvitelli”, Via Luciano Armanni 5, 80138 Naples, Italy; mascolopasquale2@gmail.com (P.M.); pierluca.zangani@unicampania.it (P.Z.); bruno.dellapietra@unicampania.it (B.D.P.); carlopietro.campobasso@unicampania.it (C.P.C.); 2 Unit of Legal Medicine, AORN “San Giuseppe Moscati”, Contrada Amoretta, 83100 Avellino, Italy; medicinalegale@aosgmoscati.av.it; 3 Unit of Infectious Disease, AORN “San Giuseppe Moscati”, Contrada Amoretta, 83100 Avellino, Italy; sebastianoleone@yahoo.it

**Keywords:** COVID-19, mortality, RT-PCR, chest computed tomography

## Abstract

(1) Background: All deaths that occurred in a hospital of Southern Italy (“San Giuseppe Moscati” Hospital of Avellino) with medium jurisdiction (up to 425,000 citizens approximately) in the period from 9 March to 4 May 2020 were analyzed. The primary endpoint of the study was to analyze the causes of death in the period study. Secondary endpoints included: (1) the assessment of overall mortality in the emergency period compared with the same period of the past years (2018–2019) in the jurisdiction area; (2) the assessment of the amounts of deaths with positive and negative reverse transcription-polymerase chain reaction (RT-PCR) of nasopharyngeal and oropharyngeal swabs; (3) the frequency of clinical and radiological features consistent with Covid-19 infection in negative RT-PCR cases. (2) Methods: Patients’ information and laboratory data were collected through the computerized medical record system (My Hospital, Italy) used for the clinical management of all referring patients. Epidemiological, clinical, and radiological data were reviewed along with the results of nasopharyngeal and oropharyngeal swabs. (3) Results: From 9 March to 4 May 2020, 140 deaths (87 males, 53 females) from all causes occurred in total at “San Giuseppe Moscati” Hospital, of which 32 deaths were Covid-19 related. (4) Conclusions: The excess of mortality could be higher than the one reported in the official epidemiological surveys. False negative cases can have a distorting effect on the assessment of the real mortality rate and the excess mortality. Furthermore, many who died from Covid-19 were likely never tested or they had false negative RT-PCR results. Other victims probably died from causes indirectly related to Covid-19.

## 1. Background

COVID-19 (coronavirus disease) is still a global health emergency [1,2]. It is a pneumonia caused by a novel enveloped RNA betacoronavirus [3] that has currently been named severe acute respiratory syndrome coronavirus 2 (SARS-CoV-2) [4]. This infection is characterized most frequently by fever, dry cough, and dyspnea [5,6,7,8]. The symptoms of Covid-19 infection commonly appear after an incubation period of approximately 5.2 days [9]. However, the asymptomatic transmission is considered the Achilles’ heel of current strategies to control the infection [10].

Actually, it is still extremely difficult to estimate the total number of infected people, because asymptomatic patients with very mild symptoms might not be tested and, therefore, not identified [11]. In fact, the gold standard for the diagnosis of Covid-19 is by reverse transcription-polymerase chain reaction (RT-PCR) [12], but delay in the results can occur because of variability in the swabs processing [13]. The total positive rate of RT-PCR for throat swab samples amounts to about 30% to 60% at initial evaluation due to the limitations of sample collections and transportation and kit performance [12,14]. Therefore, negative nasopharyngeal and oropharyngeal swab does not rule out Covid-19 infection [15].

The chest computed tomography (CT) can have a relevant role in cases with negative swabs. Chest CT scan shows peculiar features in almost all Covid-19 patients, including ground-glass opacities (GGOs), multifocal patchy consolidation, and/or interstitial changes with a peripheral distribution [16]. Chest radiological pattern was found to be useful in confirming the Covid-19 diagnosis [17,18]. However, atypical or negative chest CT findings do not exclude a Covid-19 infection, especially in the first three days, just because chest imaging may also lead to both false-negative and false-positive results [19].

The purpose of this research study is to analyze all deaths that occurred in a hospital of Southern Italy (“San Giuseppe Moscati” Hospital of Avellino) with medium jurisdiction (up to 425,000 citizens, approximately) in the period from 9 March 2020 to 4 May 2020. This is the period of pandemic lockdown in Italy during which the government imposed a national quarantine, restricting the movement of the population except for necessity, work, and health circumstances.

Avellino is located in the Campania region that was less affected by Covid-19 compared with Lombardy region in Northern Italy [20]. However, the real amount of deaths Covid-19 related could be even higher due to doubts raised about the magnitude of the infectious disease on our small community. Although, at the beginning of May, the Campania region comprised 1.25% of all Covid-19 related deaths (336 out of 29,079 in total), the Italian National Institute of Statistics (ISTAT) reports an excess mortality from coronavirus pandemic of 26,350 deaths in March and 16,283 in April all over the country [21]. These deaths are those above the average number of deaths that occurred in the previous years from 2015 to 2019 in Italy. The excess mortality was mostly due to males in the age range of 60–69 years (95%) followed soon after by those in the age range of 70–79 years (80%) and in the age range of 80–89 (57%). According to the ISTAT report [21], in Italy, the excess mortality from Covid-19 was approximately of 49.4% in March, 36.6% in April, and 3.9% in May for territories with high risk of infection. The excess mortality of Southern Italy was of 5.5% in March and 4.0% in April, which is relatively low if compared with the excess mortality of Northern Italy (96.4% in March, 71.7% in April and 3.2% in May).

Secondary endpoints of the study are: (1) the assessment of overall mortality in the emergency period compared with the same period of the past years (2018–2019) in the jurisdiction area; (2) the assessment of the amounts of deaths with positive and negative RT-PCR of nasopharyngeal and oropharyngeal swabs; (3) the frequency of clinical and radiological features consistent with Covid-19 infection in negative RT-PCR cases.

## 2. Materials and Methods

### 2.1. Subjects and Subgroup Formation

From 9 March 2020 to 4 May 2020, 140 deaths (87 males, 53 females) from all causes occurred in total at “San Giuseppe Moscati” Hospital, a 572-bed hospital located in Avellino (Italy). Patients’ information and laboratory data were collected through the computerized medical record system (My Hospital, Italy) used for the clinical management of all referring patients. Epidemiological, clinical, and radiological data were reviewed along with the results of nasopharyngeal and oropharyngeal swabs. The number of deaths at the “San Giuseppe Moscati” Hospital has been compared with the overall mortality occurred in the past years 2018–2019. No nosocomial outbreak of infection in the hospital was recorded at the same time. Data collection and analysis were approved by the Medical Ethics Committee of the “San Giuseppe Moscati” Hospital in accordance with international and institutional ethics guidelines as well as Italian legislation dealing with data protection.

### 2.2. Radiological Investigation

Image analysis was performed by one radiologist who was not aware of the RT-PCR results. The epidemiological history and the clinical symptoms (fever and/or dry cough) were the only data available for the radiologist. Radiological patterns of chest CT scan were then classified as typical or atypical for Covid-19 according to international radiological guidelines [13,22].

GGOs, particularly on peripheral and lower lobes, multifocal patchy consolidation, and crazy paving, were considered typical Covid-19 radiological patterns [18]. Atypical CT findings have included pleural or pericardial effusion, cavitation, and lymphadenopathies [23].

All CT images were obtained with CT systems (Siemens SOMATOM Sensation 16) with patients in supine position. The main scanning parameters were as follows: tube voltage, 100 kVp; automatic tube current modulation; tube current, 30–70 mAs; pitch, 0.99–1.22 mm; matrix, 5,123,512; slice thickness, 3 mm; and field of view, 350 mm 3350 mm. All images were then reconstructed with a (16 slices) slice thickness of 0.625–1.250 mm with the same increment.

### 2.3. Molecular Test

Among the 140 deaths, 108 nasopharyngeal and oropharyngeal swabs were carried out in total. Most of the swabs were taken from patients admitted to the hospital, except in 9 cases, where the throat samples were collected soon after death and, in a single case, 24 h after death. All the post mortem swabs gave negative RT-PCR results.

The RT-PCR assays were performed by using TaqMan One-Step RT-PCR Kits (Shanghai Huirui Biotechnology (Shanghai, China) or Shanghai BioGerm Medical Biotechnology (Shanghai, China)), which have been approved for use by the China Food and Drug Administration. The sensitivity of the RT-PCR diagnostic test has been reported to be 0.777 (95% CI: 0.715, 0.849), while the specificity was 0.988 (95% CI: 0.933, 1.000) with a conspicuous rate of false negative results, likely missing between 15% and 29% of patients with Covid-19 [24].

Among the 108 throat swabs, 76 cases had negative RT-PCR for Covid-19 (study group #1) and, in 32 deaths, RT-PCR were positive (study group #2). These 32 deaths were considered to be cases suffering from Covid-19 because laboratory confirmation occurred according to WHO criteria for the diagnosis of Covid-19 [2]. For the additional 32 deaths out of 140 total, no swabs were performed, as the cause of death was something other than Covid-19. These 32 deaths (study group #3) were excluded from the present survey, as the cause of death was certainly something other than Covid-19. They were mostly natural deaths due to diseases of the cardiovascular system (myocardial infarction, stroke, etc.) and the nervous system (brain cancer, muscular dystrophy, etc.) and some traumatic deaths due to traffic injuries, accidental falls, poisoning, etc.

**Figure 1:**
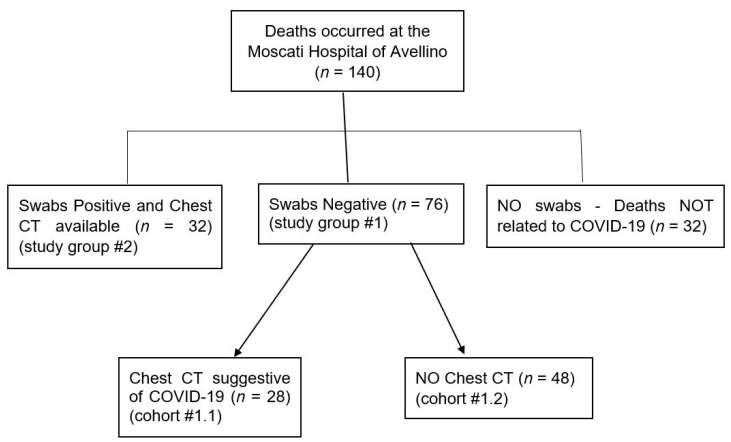
The flow diagram depicting the study groups.

Figure 1 reports the flow diagram depicting the study groups. Among the 76 cases with negative RT-PCR results (sample study #1), 28 deaths (cohort #1.1) showed chest CT features consistent with Covid-19 disease. In this cohort #1.1, the 10 post mortem swabs are included. For 48 patients out of 76 (cohort #1.2), no chest CT was available.

### 2.4. Statistical Analysis

A statistical analysis was performed using the χ2 test to compare the typical CT findings and the RT-PCR results in the study group. A *p*-value < 0.05 was considered statistically significant.

## 3. Results

In the national lockdown period, 32 deaths from coronavirus out of 53 occurred at the “San Giuseppe Moscati” Hospital in Avellino (Italy).

**Figure 2:**
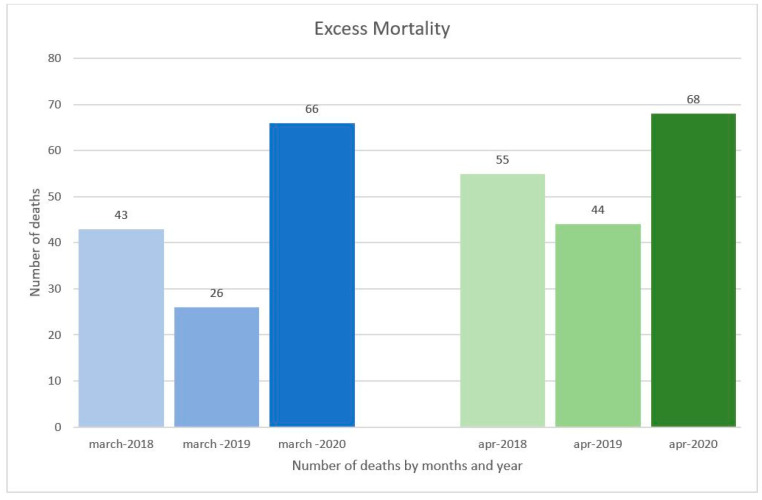
The excess mortality from Covid-19 in March and April 2020 in comparison with the number of deaths reported in the same period in 2018 and 2019 at the “San Giuseppe Moscati” Hospital in Avellino (Italy).

In Figure 2, it is shown the excess mortality from Covid-19 in comparison with the number of deaths reported in 2018 and 2019. According to the hospital register, the total number of deaths reported in March and April 2020 was 66 and 68, respectively, against an average of 34.5 cases that occurred at the same period in the previous years of 2018 and 2019. Therefore, the excess mortality reported at the “San Giuseppe Moscati” Hospital of Avellino was higher than the one observed in Campania but lower than that of the territories in the Northern Italy at high risk of infection and mortality from coronavirus [21].

The present retrospective survey was performed on 140 fatal cases that occurred in the “San Giuseppe Moscati” Hospital of Avellino from 9 March to 4 May 2020.

**Figure 3:**
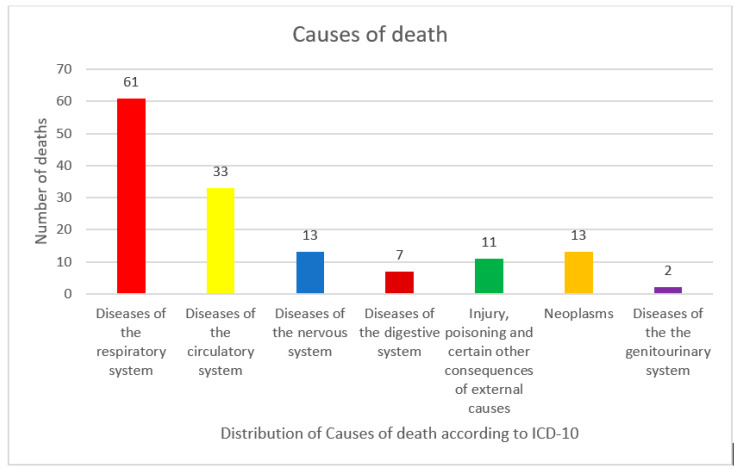
The distribution of causes of death among the 140 hospitalized victims.

According to the International Classification of Disease (ICD-10), causes of death involving the respiratory system were the most represented (61 cases out of 140) followed soon after by those affecting the circulatory system (33 cases) and the nervous system (13 cases). The distribution of causes of death is shown in Figure 3.

Victims were mostly males (87 out of 140 in total—62%) with an age range between 44 and 97 years old (mean age of 75 years, median age of 78 years). The cause of death in patients with positive and negative RT-PCR results but associated with CT scan features suggestive of Covid-19 (32 + 28 cases) was assessed as pneumonia in 60 cases out of 140 totally.

All 32 patients with positive RT-PCR (group #2) showed clinical and/or radiological patterns consistent with Covid-19 disease and, therefore, these cases were confirmed as Sars-CoV-2 related deaths. The main chest CT findings were represented by GGOs alone in 18 cases out of 32 (56%), multifocal patchy consolidations associated with GGOs in 4 deaths (12%), and crazy paving associated with GGOs in 10 deaths (32%). Therefore, the GGOs were found in all 32 victims (group #2). Most of these victims were males (25 out of 32) with an age range between 55 and 94 (mean age of 75 years, median age of 73 years). All the 32 victims (group #2) had comorbidity mostly represented by hypertension in 17 cases out of 32 (53%) followed by cardiovascular diseases (12 patients out of 32—38%), chronic obstructive pulmonary disease (COPD) in 9 cases (28%), and diabetes in 6 cases out of 32 (19%).

**Figure 4:**
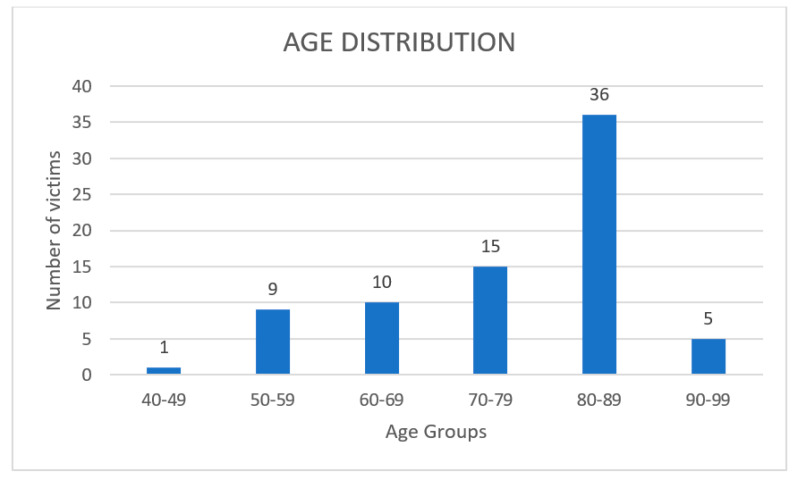
Age distribution of the study group #1.

Study group #1 (76 deaths with RT-PCR negative to Covid-19) was represented by 45 males and 31 females, with a mean age of 76 years (age distribution shown in Figure 4).

Among these 76 negative cases, 28 deaths (cohort #1.1) presented clinical (fever, cough or dyspnea) and radiological findings suggestive of Covid-19 (ground-glass, consolidation, crazy paving), while in the remaining 48 deaths (cohort #1.2), no chest CT was available. For all the 76 victims, the cause of death was assessed as something other than Covid-19, although in most of the victims, severe diseases of respiratory and circulatory systems occurred (58 out of 76). However, based on the negative results of the swabs, these diseases were considered non-COVID-19 related.

The causes of deaths of 48 patients (cohort #1.2) with negative RT-PCR and no CT-chest available nor clinical symptoms Covid-19 related were mainly due to diseases of circulatory system (20 out of 48), respiratory system (10 out of 48), digestive system (2 out of 48), neoplasms (7 out of 48), nervous system (1 out 48), infectious disease other than coronavirus (3 out of 48), blunt trauma and other injuries (3 out of 48), and diseases of genitourinary system (2 out of 48).

The retrospective survey of the 28 cases belonging to cohort #1.1 (deaths with negative RT-PCR but with severe respiratory symptoms) raised initial doubts about the relationship with Sars-CoV-2 mainly because of the typical imaging features of Covid-19 pneumonia observed in chest CT. Therefore, a special focus has been performed on this cohort of 28 deaths (15 males, 13 females; median age 79 years, and age range between 65 and 87 years) with negative RT-PCR tests but with clinical and radiological imaging consistent with Covid-19 infection. According to recommendations developed for cases with negative RT-PCR but also suggestive clinical and radiological findings [13], second swabs were taken in 18 patients out of 28 in total. They were collected mostly ante-mortem but also post-mortem in 10 cases. All the RT-PCR results were again negative.

The main chest CT findings were represented by GGOs alone in 3 cases out of 28 (11%), multifocal patchy consolidations associated with GGOs in 9 deaths (32%), and crazy paving associated with GGOs in 16 deaths (57%). Therefore, GGOs were again the most common radiological abnormalities found in all the 28 victims with negative RT-PCR (cohort #1.1), although in 10 cases out of 28 in total (36%), they were associated with non-typical chest CT findings such as pleural or pericardial effusion, cavitation, and lymphoadenopathies. Statistically significant difference (*p* = 0.001, χ^2^ = 13.82, df = 2) was found in the comparison between the RT-PCR results and typical findings (Table 1). Therefore, 18 cases out of 28 in total (64%) showing typical findings suggestive of Covid-19 could be potentially classified as deaths from Sars-CoV2.

**Figure 5:**
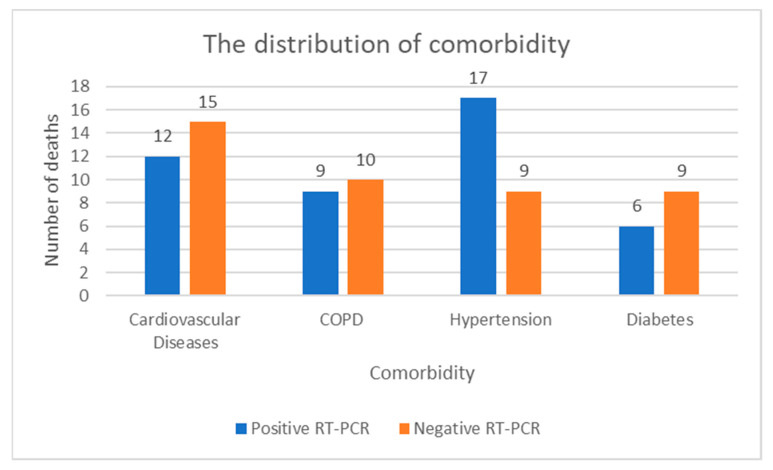
The distribution of comorbidity among the 32 victims with positive RT-PCR (study group #2) and the 28 deaths with negative RT-PCR (cohort #1.1).

Regarding the clinical findings that occurred among these 28 cases (cohort #1.1) with negative RT-PCR, the most common symptom was dyspnea (64%). The average duration of hospitalization was 8 days, and five patients (18%) were admitted to the intensive care unit (ICU) and underwent invasive mechanical ventilation. All the victims had severe comorbidity mostly represented by cardiovascular diseases in 15 out of 28 (54%), followed by COPD in 10 cases out of 28 (35%) and hypertension and diabetes both in 9 cases out of 28 (32%). The distribution of comorbidity among the 32 patients with positive RT-PCR (study group #2) and the 28 victims with negative RT-PCR (cohort #1.1) is summarized in Figure 5.

## 4. Discussion

Case-fatality statistics in Italy are based on defining Covid-19 related deaths as those that occurred in patients who test positive for SARS-Cov-2 via RT-PCR, independently from preexisting diseases that may have caused death [25]. In our sample study, 32 deaths (study group #1) out of 140 in total were assessed due to Covid-19 based on positive RT-PCR and suggestive clinical and/or radiological findings. No causes of deaths other than Covid-19 occurred in this cohort of 32 Covid-19 deceased patients. The cause of death for 76 patients (study group #2) with negative RT-PCR was assessed as something other than Covid-19 and for 28 victims (cohort #1.1) who presented clinical and radiological findings consistent with Covid-19 infection. They were considered deaths not Covid-19 related just because they had negative RT-PCR results, even after a second swab taken in 18 patients out of 28 in total. The results of this retrospective survey suggest an underestimation of the effects of the Covid-19 disease on the health of a medium community such as the Avellino’s one.

In this retrospective survey, the positive rate of RT-PCR assay was 30% (32 cases out of 108 swabs in total), although previous studies performed on a large sample of patients reported a greater sensibility ranging between 30 to 70% [12,14,26].

RT-PCR sensitivity depends mostly on the type of test and assay, the quality of the throat swab, and the viral load [13]. RT-PCR testing can be influenced by sampling operations, specimen source (upper or lower respiratory tract), sampling timing (different period of disease development [14,27,28], and performance of detection kits [12]. Negative results of nasopharyngeal and oropharyngeal swabs do not rule out Covid-19 disease for sure [15]. It must be taken into account that swabs can also have insufficient stability and relatively long processing time affecting RT-PCR results [12].

Therefore, false negative results of RT-PCR cannot be ignored. This could be the case of the 28 victims with negative RT-PCR (cohort #1.1). Unfortunately, SARS-Cov-2 serology was not currently available during the reference period of the first wave of the pandemic outbreak. In fact, the serologic assays to detect antibodies against SARS-CoV-2 are of great interest in cases of negative RT-PCR [29]. High levels of IgM and IgG can be detected from the second week of symptom’s onset, since IgM can be positive from the fourth day and IgG after 8 days. [30,31]. The accuracy of some serological tests is near 100% when samples are taken 20 days after infection [31].

Due to the limitations of detection kits available, a Covid-19 disease for the 28 cases of cohort #1.1 cannot be excluded, but the hypothesis that these deaths could be Covid-19 related is not evidence-based. According to literature [17,32], negative RT-PCR but typical chest CT features can be highly suggestive of Covid-19, particularly in areas with high prevalence of Covid-19. In fact, chest CT findings may be a more reliable, practical, and rapid method to assess a Covid-19 disease [12] and could have a role especially in cases with negative swabs. CT findings suggestive of Covid-19 were also observed in patients with negative RT-PCR results along with specific clinical symptoms [12].

Small-scale studies have demonstrated that the current RT-PCR testing has limited sensitivity [12], while chest CT may reveal pulmonary abnormalities consistent with Covid-19 in patients with initial negative RT-PCR results [17,33,34].

According to international radiological guidelines [22], these radiological abnormalities are mostly represented by GGOs, particularly on peripheral and lower lobes with/without crazy paving sign and multifocal patchy consolidation. They were found to be a typical radiological pattern that could be used to diagnose Covid-19 infection in patients with high clinical suspicion and negative initial RT-PCR [17,18]. CT abnormalities suggesting Covid-19 infection have been observed in up to 29.4% of patients with initially negative RT-PCR [26].

These radiological abnormalities are characterized by an increased lung opacity commonly found in Covid-19 related deaths, but they cannot be considered specific for Covid-19, as they can be also associated with other viral and bacterial infections. Non-typical CT findings have included pleural effusion, masses cavitation, and lymphadenopathies [23]. In fact, accumulation of pleural fluid is not a specific disease but the sign of underlying pathology such as congestive failure, cancer, pulmonary embolism, and pneumonia due to causes other than Covid-19 [35]. Pleural effusion can occur in Covid-19 related pneumonia but it has been found to be uncommon in previous studies. It can be a possible radiological finding observed with disease progression, reported in 3–4% of patients with Covid-19 pneumonia [36,37]. This is the reason why it has been classified as a non-typical CT finding compared to GGOs and consolidation with peripheral distribution by international radiological guidelines [13,22].

In cohort #1.1, 18 cases out of 28 with negative RT-PCR showed typical CT findings suggesting probable Covid-19 infection [18] compared to atypical CT findings for Covid-19 pneumonia observed in 10 cases. For these latter 10 cases, it is worth mentioning that atypical chest CT findings do not exclude a Covid-19 infection, especially in the first three days, just because chest imaging may also lead to both false-negative and false-positive results [19].

According to the current state of the art, all the 28 patients with negative RT-PCR (cohort #1.1) were classified to be suffering of pneumonia not COVID-19 related, although clinical and radiological chest CT findings were suggestive for COVID-19 in 18 out 28 cases. It is our opinion that diagnosis of COVID-19 cannot be excluded for these 18 victims who tested negative for Covid-19. Serological platforms are actually very useful to reveal infections based on the SARS-Cov-2-specific antibody responses, and they could be of help for the Covid-19 diagnosis.

Therefore, the discrepancy between typical CT findings and RT-PCR results is not new in literature [17,34,38], and it could be related to factors affecting the performance of these investigations according to their sensitivity and timing of examinations [27]. Unfortunately, false-negative and false-positive results of RT-PCR and CT-scan can occur, especially in the early stages of the disease, and they still need to be better identified and investigated. In fact, RT-PCR allows the identification of the infection in the early phase, even if there is a period of a few days, called the “window” period, where the subject is negative [28,38]. False negative RT-PCR results can be related to inadequate viral material (both quality and volume) at the time of specimen collection or technical issues during nucleic acid extraction [28,34]. In a systematic review of 919 patients [39], CT scan can also provide atypical findings, as the greatest severity of imaging has been reported around 10 days after symptom onset in the early stage of the disease. Imaging features can also vary depending on the disease stage during follow-up and timing of scanning, drug interventions, immunity status, and patient’s age [17].

The novel coronavirus can be particularly harmful to people with pre-existing chronic conditions, which are common, especially among the elderly. In fact, patients belonging to cohort #1.1 had an age ranged between 65 to 87 years old with a median age of 79 years. A similar median age (75 years) was found in patients with positive RT-PCR (study group #2) with an age range between 55 and 94 years. Both study samples (group #2 and cohort #1.1) showed severe comorbidity represented mostly by hypertension and other cardiovascular diseases (chronic heart failure, dilatative cardiomyopathy secondary to myocardial infarction, atrial fibrillation, etc.), COPD, and diabetes. According to literature, these pre-existing conditions are relatively common in Covid-19 related deaths, and they correlate with poor clinical outcomes [4]. They may be serious risk factors for severe patients among which, in particular, hypertension and other heart disease pathologies are usually found in elderly population [40,41].

All causes of mortality can be interpreted as an approximation of the health status [20], and the full implications of the COVID-19 pandemic will be understood when more information on its pathogenesis and mechanisms of death is available. Due to the limited information available, most issues related to the dynamics of the COVID-19 disease have still significant uncertainties [42].

According to the definition for deaths due to COVID-19 provided by WHO in its guidelines [43], “a death due to Covid-19 should be resulting from a clinically compatible illness, in a probable or confirmed Covid-19 case, unless there is a clear alternative cause of death not related to Covid-19”. Therefore, the cohort of 28 patients (cohort #1.1) suffering of pneumonia but with inconclusive testing for SARS-Cov-2 could be considered at least suspect cases, especially in cases with chest CT typical findings for COVID-19. Thus, false negative multiple RT-PCR results can be diagnostically challenging [17]. It is our suspect that Covid-19 mortality is currently underestimated due to the high rate of false negatives in the population tested for Covid-19, the lack of sensitivity of RT-PCR specimens, and factors affecting RT-PCR results (e.g., inappropriate sampling from upper or lower respiratory tract, inappropriate timing of sampling with a delay between administering and processing the test, low performance of detection kits) [28]. It is also worth mentioning that, in the early stage of disease, chest imaging can also provide atypical findings so that international radiological guidelines from the Centers for Disease Control (CDC) [44], the American College of Radiology (ACR) [45], and the British Society of Thoracic Imaging (BSTI) [22] do not recommend CT scans to diagnose Covid-19.

## 5. Conclusions

This study has several limitations, including small samples size and the fact that it focuses on deaths and not on all cases of Covid-19. However, according to literature, doubts can be raised about the accuracy of confirmed case and death counts, indicating a substantial underestimation of the magnitude of the burden of Covid-19 [20]. The excess of mortality could be higher than the one reported in the official epidemiological surveys. The true death rate from Covid-19 is strongly limited by the lack of reliable testing methods and full knowledge of this disease [42]. False negative cases can have a distorting effect on the assessment of real mortality rate and excess mortality. Furthermore, many who died from Covid-19 were likely never tested or they had false negative RT-PCR results or false negative chest imaging in the first three days. Other victims probably died from causes indirectly related to Covid-19 [20] and in these cases, autopsy can represent a reliable tool in the differential diagnosis [27].

## Figures and Tables

**Table 1:** Comparison between the reverse transcription-polymerase chain reaction (RT-PCR) results and typical CT findings. GGO: ground-glass opacities.

RT-PCR Results	GGOs	GGOs + Crazy Paving	GGOs + Multifocal Patchy Consolidations	*p*-Value
Positive RT-PCR, n. 32 (53.3%)	18 (56%)	10 (32%)	4 (12%)	0.001
Negative RT-PCR, n. 28 (46.7%)	3 (11%)	16 (57%)	9 (32%)	
Total n. 60 (100%)	21 (35%)	26 (43.3%)	13 (21.7%)	

## Data Availability

The data presented in this study are available on request from the corresponding author. The data are not publicly available due to privacy restriction.
